# A Practical and Automated Approach to Large Area Forest Disturbance Mapping with Remote Sensing

**DOI:** 10.1371/journal.pone.0078438

**Published:** 2014-04-09

**Authors:** Mutlu Ozdogan

**Affiliations:** Department of Forest and Wildlife Ecology & Center for Sustainability and the Global Environment, University of Wisconsin – Madison, Madison, Wisconsin, United States of America; NASA Jet Propulsion Laboratory, United States of America

## Abstract

In this paper, I describe a set of procedures that automate forest disturbance mapping using a pair of Landsat images. The approach is built on the traditional pair-wise change detection method, but is designed to extract training data without user interaction and uses a robust classification algorithm capable of handling incorrectly labeled training data. The steps in this procedure include: i) creating masks for water, non-forested areas, clouds, and cloud shadows; ii) identifying training pixels whose value is above or below a threshold defined by the number of standard deviations from the mean value of the histograms generated from local windows in the short-wave infrared (SWIR) difference image; iii) filtering the original training data through a number of classification algorithms using an n-fold cross validation to eliminate mislabeled training samples; and finally, iv) mapping forest disturbance using a supervised classification algorithm. When applied to 17 Landsat footprints across the U.S. at five-year intervals between 1985 and 2010, the proposed approach produced forest disturbance maps with 80 to 95% overall accuracy, comparable to those obtained from traditional approaches to forest change detection. The primary sources of mis-classification errors included inaccurate identification of forests (errors of commission), issues related to the land/water mask, and clouds and cloud shadows missed during image screening. The approach requires images from the peak growing season, at least for the deciduous forest sites, and cannot readily distinguish forest harvest from natural disturbances or other types of land cover change. The accuracy of detecting forest disturbance diminishes with the number of years between the images that make up the image pair. Nevertheless, the relatively high accuracies, little or no user input needed for processing, speed of map production, and simplicity of the approach make the new method especially practical for forest cover change analysis over very large regions.

## Introduction

Rapid assessment of forest disturbance is an important source of information for studies ranging from global environmental change to local forest management planning [1], [2]. When viewed from a disturbance perspective, changes in forested areas are also important for understanding fluxes of carbon and water between the biosphere and the atmosphere. For example, although North American forests have been viewed as a net carbon sink, the magnitude of the sink is uncertain and requires careful assessment of land use history including harvest [3], [4], [5], [6], [7]. From a forest management perspective, the age and composition of forests are determined in part by harvest history, and land management influences biodiversity and other ecosystem services in forested areas.

Remote sensing, either alone or in combination with field studies, has made great contributions to documenting land use history in forested landscapes [8]. In particular, the Landsat system has been the workhorse for characterizing many forest changes given its long-standing archival imagery, dedicated acquisition strategy, well-calibrated observations, and high spatial resolution which provides the necessary detail to document fine-grain changes. The literature on remote sensing-based change detection studies in forested areas is rich with a wide range of case studies, methods, and applications in virtually every type of environment [9], [10], [11], [12], [13], [14], [15].

In recent years, a variety of new methods have been developed to process and extract forest disturbance information, including semi-automated methods [16], [17], [18], [19], [20], [21]. These methods rely on the availability of relatively dense acquisitions (annual or biennial) of image data, which may exist only in a few places (e.g. the United States), despite the recent opening of the Landsat archive [8]. In other locations, characterization of land cover change still occurs at relatively sparse temporal intervals [9], [13], relying on pairs of images from different years. In these locations, image acquisition strategies coupled with issues related to cloud cover and the Landsat 7 Scan-Line-Corrector (SLC)-Off problem preclude image analysis that requires dense time series. In regions with limited temporal coverage, other approaches are still needed that are sufficiently generalizable and repeatable to document forest cover changes through time.

In this paper, I revisit the traditional pair-wise change detection method for forest disturbance mapping and provide a set of procedures that automate the process of image-to-image information translation. The methods presented here rely on the distinct spectral signature associated with forest disturbance captured in a pair of Landsat images. The approach also relies on the ability to automatically capture this signal without user interaction and use it in a robust supervised classification algorithm that can handle incorrectly labeled training data. The method is essentially a supervised classification exercise, but eliminates the need for manual image interpretation for extracting training data. It is specifically designed to work in situations where dense time stacks of Landsat imagery are not available and the user is forced to use pair-wise image analysis [20]. If dense time series of data are available, the proposed method is also applicable by using a sequence of image pairs.

## Materials and Methods

**Figure 1:**
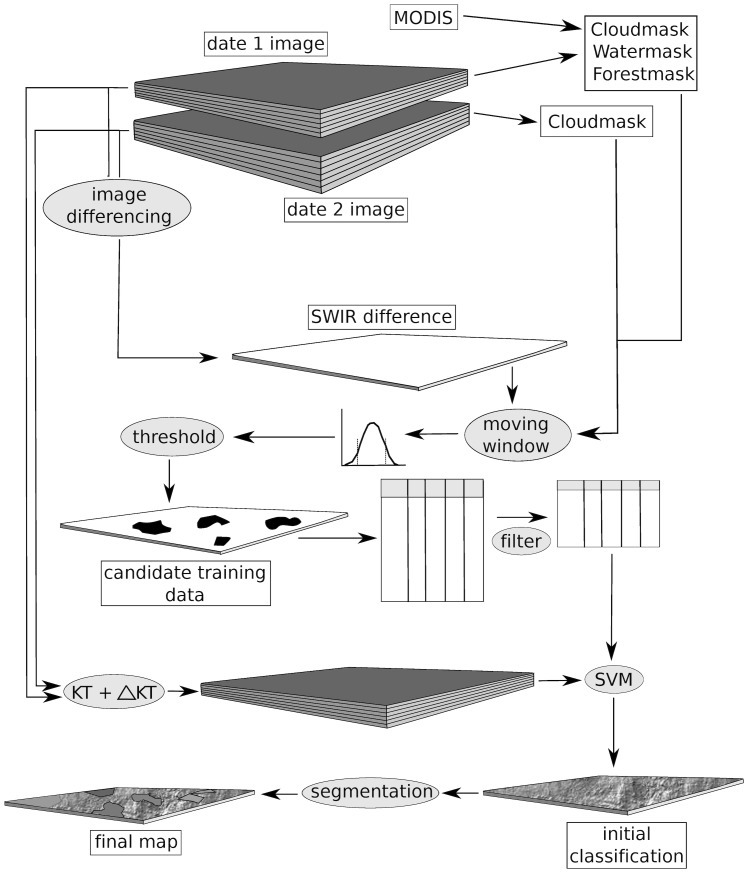
Graphical representation of the steps involved in the automated change detection method. Ellipses with light gray color represent processes, rectangles with back fill represent results, and the elongated squares represent single- or multiple-layer image sources.

The methodology includes the following steps: a) preprocessing of the Landsat image pairs by masking all clouds, cloud shadows, and non-forested areas such as water and agriculture (sections 2.2, 2.3, and 2.4); b) automated extraction of training data from local windows using the Landsat shortwave-infrared (SWIR) band difference image with local windows (section 2.5); c) removal of incorrectly labeled instances in the training data through *n*-cross validation (section 2.6); d) classification of disturbed areas using a supervised classification algorithm (sections 2.7 and 2.8); and e) per-pixel to per-region translation of classification results using segmentation (section 2.9). These steps are illustrated in Figure 1 and described below in further detail.

### Data

To test the automated method, I used 17 Landsat footprints defined by the World Reference System 2 (WRS2) path/row structure (Table 1). These footprints were chosen to a) maximize areal coverage; and b) represent a large variety of forested ecosystems. For each footprint, a total of five image pairs acquired by the Landsat Thematic Mapper (TM) and the Enhanced Thematic Mapper Plus (ETM+) sensors at five-year intervals between 1985 and 2010 (1985–1990; 1990–1995; 1995–2000; 2000–2005; and 2005–2010) were used. The twice-per-decade interval reflects the tradeoff for the detection of disturbance/regrowth and the limitation of available remotely sensed data to detect these changes. Due to data limitations, images from plus/minus one year of the nominal target year were used when assembling the image dataset.

**Table 1:** Dates of test images used in the analysis.

path/row	1985	1990	1995	2000	2005	2010
11/28	7/1/85	7/2/91	6/27/95	6/22/99	7/8/05	6/17/09
13/30	9/1/85	9/2/91	8/30/96	9/10/00	9/8/05	8/18/09
15/36	5/26/85	5/8/90	5/6/95	5/3/00	5/4/06	4/29/10
16/31	7/23/86	8/3/90	8/1/95	8/1/01	7/11/05	7/6/09
17/33	9/13/85	8/29/91	8/24/95	9/6/00	8/3/05	9/2/10
17/38	11/13/84	11/14/90	11/14/96	10/27/01	11/23/05	10/23/11
19/36	5/19/84	5/7/91	5/4/96	5/15/00	5/13/05	5/27/10
21/29	9/6/84	8/25/91	9/18/94	9/18/00	9/13/04	8/29/10
21/38	6/24/86	6/19/90	6/17/95	6/17/01	5/27/05	5/25/10
24/38	11/1/85	10/30/90	10/30/96	10/25/00	10/23/05	11/6/10
25/28	9/18/84	9/16/89	9/1/95	8/29/00	8/27/05	9/7/09
28/27	8/28/86	9/5/89	9/19/94	9/17/99	9/4/06	8/17/11
34/33	7/2/85	7/13/89	6/25/94	7/1/02	7/6/04	6/21/10
42/27	7/26/85	8/6/89	8/9/96	8/7/01	7/20/06	7/15/10
43/33	9/16/84	9/1/90	9/15/95	9/12/00	9/7/04	9/24/10
46/27	8/23/85	9/9/91	8/21/96	8/16/00	7/29/05	7/30/11
46/30	8/4/84	8/8/91	8/3/95	8/19/01	7/29/05	7/24/09

The selected images were ordered from the US Geological Survey (USGS) as terrain corrected, thus no geometric normalization was required. Each image in the time stack was chosen to contain minimal or no cloud contamination and was acquired during the peak-growing season. It was difficult to completely avoid cloud issues so a robust cloud and cloud shadow screening algorithm was used. In situations where an optimal image pair match from peak growing season was not available, non-ideal images were selected but this decision came with a tradeoff in classification accuracy (see below). No ETM+ images acquired after May 2003 were used in image pairs to avoid dealing with data gaps resulting from the SLC problem, although the proposed methodology could be revised to accept the SLC-Off images.

### Cloud and Cloud Shadow Masking

The biggest challenge that cloud and cloud shadows cause in forest disturbance analysis is the change in brightness values between image dates that resembles the spectral changes associated with forest disturbance. To avoid this problem, and the misclassification that results, I applied a robust cloud and cloud shadow detection algorithm developed by [22]. This method uses a series of rules based on calculated probabilities of temperature, spectral variability, and brightness, using Top of Atmosphere (TOA) reflectance and Brightness Temperature (BT) as inputs. The clouds and cloud shadows are treated as 3D objects determined via segmentation of the potential cloud layer and an assumption of a constant temperature lapse rate. The solar illumination and sensor view angles are used to predict possible cloud shadow locations and select the one that has the maximum similarity to cloud shape and size.

### Land–water Masking

The constant changing nature of inland water bodies also pose problems for automatic forest change detection as the expansion and contraction of lakes and rivers between image dates cause darkening or brightening of image pixels. To identify and remove water pixels, I used a procedure proposed by [19] that is based on known spectral properties of water bodies such as low reflectance in the SWIR (2.1–2.8 *μm*). Pixels are labeled as water if they have low SWIR reflectance (less than 25%) and have either a decreasing trend in reflectance values from the visible to the infrared bands or have normalized difference vegetation index (NDVI) values less than 0.3, or both.

### Forest/Non-forest Masking

The purpose of this step is to identify potentially forested areas and limit the application only to those locations. Note that while it is possible to use existing land cover datasets, such as the US National Landcover Dataset (NLCD) [23], map accuracy limitations and the absence of this data set outside the US make its use impractical for global applications. To identify pixels that are likely to be forested I used the approach proposed by [16], which is based on known spectral properties of forest canopies. Due to the dark nature of red light in forested areas, this approach uses the location of the dark peak in histograms extracted from local image windows of the Landsat red band (Band 3). Referred to as the “forest peak”, the location of this peak is used as a threshold to separate forested pixels from their non-forested counterparts.

### Automatic Extraction of Training Data for Forest Harvest

In this research, the process of extracting training data is accomplished by identifying thresholds in histograms created from local SWIR-band image windows. The SWIR portion of the electromagnetic spectrum is widely recognized as containing the most useful information for detecting changes in forested areas [24]. Given a pair of SWIR reflectance images from two different dates, subtraction of the second image date from the first date will yield large negative reflectance values in disturbed forested areas. This is because SWIR reflectance is often low in mature forests and high in disturbed areas. In contrast, pixels representing forest recovery/regrowth exhibit a large increase in reflectance values between the two dates. The overall distribution of the SWIR reflectance difference image is Gaussian with a mean value near zero, primarily because the majority of the pixels between the two dates exhibit no change and form the bulk of the distribution. In contrast, areas that experienced forest disturbance or recovery are in the negative (removal) and positive (regrowth) ends of the distribution. In this research, the crucial part of the analysis is to automatically identify the threshold values that separate change from non-change locations in this histogram. Any deviation of the mean from zero in the Gaussian distribution reflects the atmospheric/radiometric/phenological differences between image dates and is ignored for the purpose of this analysis. Note that in change detection studies involving supervised image classification where training data are extracted directly from the image pair to be classified (e.g. this study), image radiometric and/or atmospheric correction is not necessary [25].

The training data to identify pixels as disturbed, regrowth, and no-change were extracted using thresholds applied in local image windows of the two-date SWIR reflectance difference image. A local window is defined as a square portion of an image, usually 400 by 400 pixels in size, extracted using a moving window approach. When a local image window has sufficient proportions of disturbed, regrowth, and no change areas, a fixed number of standard deviations away from the mean is used to identify the thresholds for training data. More specifically, in the absence of water, clouds, cloud shadows, and non-vegetated surfaces – which would already be masked out – forest disturbance and regrowth pixels are identified as pixels whose value exceeds the threshold defined by 1.5 times the standard deviations in either direction. Note that while the value of the threshold changes across windows and images, the number of standard deviations used to identify the threshold is fixed across all windows and images. This number was determined empirically to balance omission and commission errors, using a large number of Landsat footprints. The purpose of using local image windows is to select an appropriate threshold that is not easily determined from global image statistics.

### Identification of Incorrectly Labeled Training Data

The automatic extraction of training data can result in labeling errors arising from the selection of inappropriate thresholds, inaccurate masks, and mis-identification of forested areas. Previous research has shown that removing mislabeled samples significantly improves predictive accuracy of the classification process [26], [27], [28], [29]. This step focuses on improving the quality of the training data by identifying and eliminating mislabeled training samples. To achieve this goal, I followed the filtering approach originally proposed by [30] in which the raw training data is passed through a number of classification algorithms through an *n*-fold cross-validation process that serve as a *filter* to remove mislabeled samples. More specifically, a number of subsets (*n*), each of which capture about 70 percent of the population, are first selected from the population randomly with replacement. Then for each of *n* subsets, the *m* algorithms were trained on the other *n* - 1 instances. The *m* resulting classifiers were then used to label each sample as either correct or mislabeled in the excluded samples. Once all of the samples tagged as mislabeled are removed, the new (i.e. filtered) training set is used as the final training input to the classification algorithm described below. An interesting feature of this approach is that it employs a consensus filter where only those samples that all of the individual classifiers tagged as mislabeled are discarded [30]. The approach is functionally equivalent to techniques for removing outliers in regression analysis where an outlier is defined as a case that does not follow the same model as the rest of the data [30].

In this implementation, I used four well-known classification algorithms: Support Vector Machines (SVM) [31], Decision Trees (DT) [32], K-Nearest neighbor (KNN) [33], and Artificial Neural Networks (NN) [34]. To ensure sufficient representation in the training process, the original training dataset was divided into a training set (70%) and a testing set (30%). For each run, the distribution of the classes in the sample set was forced to reflect the class distribution of the entire data set. At the end of the 10-fold cross-validation process, samples that were incorrectly classified by all four algorithms were identified as mislabeled and removed from the analysis. Finally, the filtered training data are provided as an input to the final SVM classifier. Note that apart from SVM, the three pattern recognition algorithms (DT, KNN, and NN) chosen for the filtering task were chosen somewhat arbitrarily. The filtering process is independent of the choice of algorithm – what matters is the consensus among algorithms when classifying a training instance into a class label.

### Support Vector Machines

Support vector machines (SVMs) are a supervised non-parametric statistical learning technique that is increasingly being used by the remote sensing community [35], [36], [37]. At the heart of an SVM training algorithm lies the concept of a linear *hyperplane* – an optimal boundary found through an iterative learning procedure that separates the training set into a discrete predefined number of classes while minimizing misclassifications errors [38], [39]. Several approaches have been developed to improve SVM predictive accuracies using multispectral remote sensing data. These include the soft margin approach [30] and kernel-based learning [40] that lead to SVM optimization, although the kernel functions often result in more expensive parameterization [41].

Prior research has identified at least three benefits of SVMs that make them particularly suitable for remote sensing applications. First, regardless of the size of the learning sample, not all the available examples are used in the specification of the hyperplane. This allows SVMs to successfully handle small training data sets because only a subset of points – the support vectors – that lie on the margin are used to define the hyperplane [36]. Second, unlike many statistical classifiers, SVMs do not make prior assumptions on the probability distribution of the data, which leads to reduction in classification errors when input data do not conform to a required distribution (e.g. Gaussian). Third, SVM-based classification algorithms have been shown to produce generalizable models from a set of input training data, eliminating the notion of overfitting [42].

To perform the SVM-based classification, I used the LIBSVM implementation that provides linear, polynomial (cubic) and radial-basis kernels [43]. This implementation includes C-support vector classification (C-SVC), ν-support vector classification (ν-SVC), distribution estimation (one-class SVM), ε-support vector regression (ε-SVR), and ν-support vector regression (ν-SVR) formulations. All SVM formulations supported in LIBSVM are quadratic minimization problems. Using the radial-basis kernel classification option, the LIBSVM required only two parameters to be defined: the kernel parameter *γ* and the cost parameter *C* [43]. Both of these parameters are data dependent and are identified separately for each footprint/date-pair combination using the grid search option over log-transformed hyper-parameters as suggested by [44].

Note that SVMs have been shown to perform well given a certain level of noise (i.e. mislabeled training data) but they are not completely impervious to outliers [45]. While a number of methods have been developed to mitigate the effects of outliers on SVMs [46], [47], [48], [49], they show only incremental improvements over standard SVM methods.

### Input Data to Classification

The primary input to the SVM classifier was the multi-temporal extension of the well-known Kauth-Thomas (KT) transformation [50], [51]. The KT indices and in particular the wetness index have been shown to be useful in mapping forest structure, condition, and disturbance in coniferous forests [52], [53], [54], [55], [56]. [57] extended the KT transform to multi-date imagery (hereafter, MKT) to arrive at three new components: change in brightness (ΔB), change in greenness (ΔG), and change in wetness (ΔW). The MKT is well-suited to forest change mapping given that: (a) MKT uses known linear transform coefficients; (b) MKT uses all of the Landsat spectral information; and (c) the MKT change in brightness, greenness, and wetness bands are physically interpretable [57]. I used a six-band image stack composed of first date’s brightness, greenness and wetness indices and MKT change indices. In this representation, the first date KT data is used to anchor the condition of forested areas prior to disturbance and the MKT indices reflect subsequent changes [58]. An additional benefit of this arrangement was reduced confusion between shifting agricultural patterns and forest clearing.

### Post-classification Processing

Raw outputs from a classification algorithm often contain significant errors when examined on a pixel-by-pixel basis. To overcome this issue, I employed a post-classification segmentation process to translate the initial per-pixel results to per-polygon outcomes (segments). To do this, the segmentation algorithm developed by [59] with a minimum mapping unit of six Landsat pixels (roughly equal to 0.5 hectares) was used. Input features to the segmentation tool included a combination of Landsat bands 3, 4, and 5 (red, NIR, and SWIR) images from the pre- and post-disturbance period (a total of six bands). Per-pixel classification results were first eroded and dilated, and converted to polygon-based outputs using a simple plurality rule. The final polygon-based classified images were more realistic and devoid of the high frequency noise present in the original per-pixel classification results. After adding in water, clouds, cloud shadows, and non-forested areas that were masked out earlier, the final map contained six categories: disturbed areas, stable forest, stable non-forest, water, clouds, and cloud shadows.

### Map Validation

An independent, stratified random sample of accuracy assessment sites was used to estimate the final map accuracy for each Landsat footprint, using traditional map accuracy measures [60], [61]. Although the final classification contained six categories, I assessed the accuracy of only two strata: *forest disturbance* and *stable forest.* For each of these categories, I randomly extracted 100 sites in the form of polygons from the segmented results. This procedure was repeated for the 17 footprints across five time periods, with a total of 17,000 samples (200 samples × 17 footprints × 5 time periods). For each sample, three analysts conducted accuracy assessment at the level of individual polygons through visual inspection of both MKT and Band 4, 5, and 3 (RGB) color composite images as well as vector overlays within Google Earth.

While it would have been desirable to use an independent, ground-validated dataset to assess the accuracy of the harvest maps, neither the data nor the resources to evaluate 17 footprints were available or logistically feasible. Assessing accuracy was especially challenging because forest harvest activity had to be evaluated for nearly three decades. Therefore, I primarily relied on imagery itself as the only consistent source of evaluation. Moreover, both [53] and [54] showed that manual interpretation of tasseled-cap transformed Landsat imagery could be as accurate for mapping forest clear-cuts as field-based datasets. Therefore, I chose to use the transformed imagery to test the agreement between harvested areas identified by our algorithm and the ones identified by the third party analyst.

## Results

### Accuracy of Classifications

**Figure 2:**
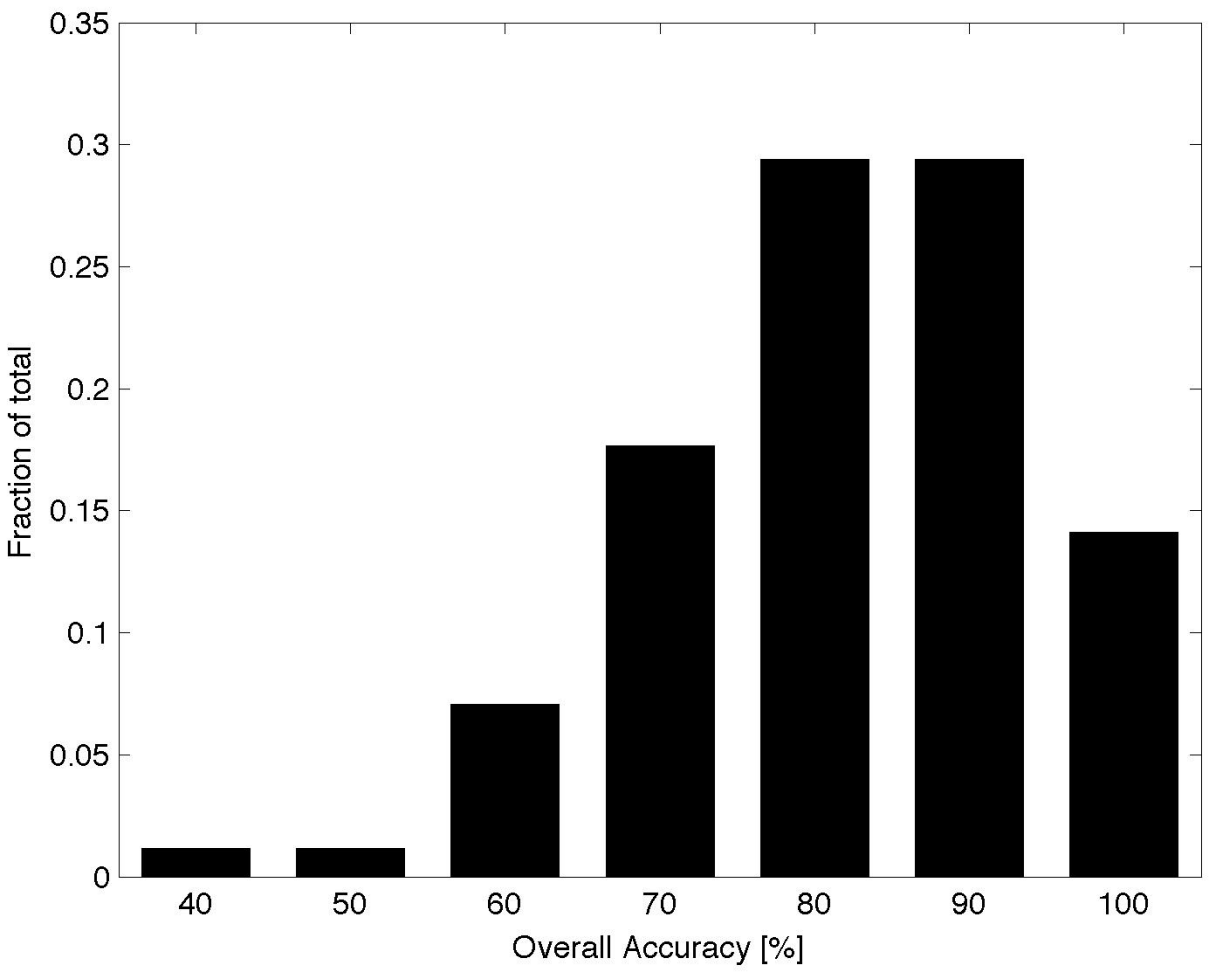
Distribution of overall accuracy values across all footprints and time periods.

The evaluation of the forest change maps derived from the automated procedure includes an assessment of the overall map accuracy, which measures the proportion of individual footprint area that is classified correctly into two categories. The overall accuracy of the forest change and stable forest classes using the automated classification across all footprints and time periods ranged from 40 to 99 percent with a mean value of 82.3±12.5 percent (N = 85) (Figure 2). These overall accuracy values are quasi-normally distributed, but are positively skewed having the bulk of the quantities between 80 and 90 percent (Figure 2).

**Figure 3:**
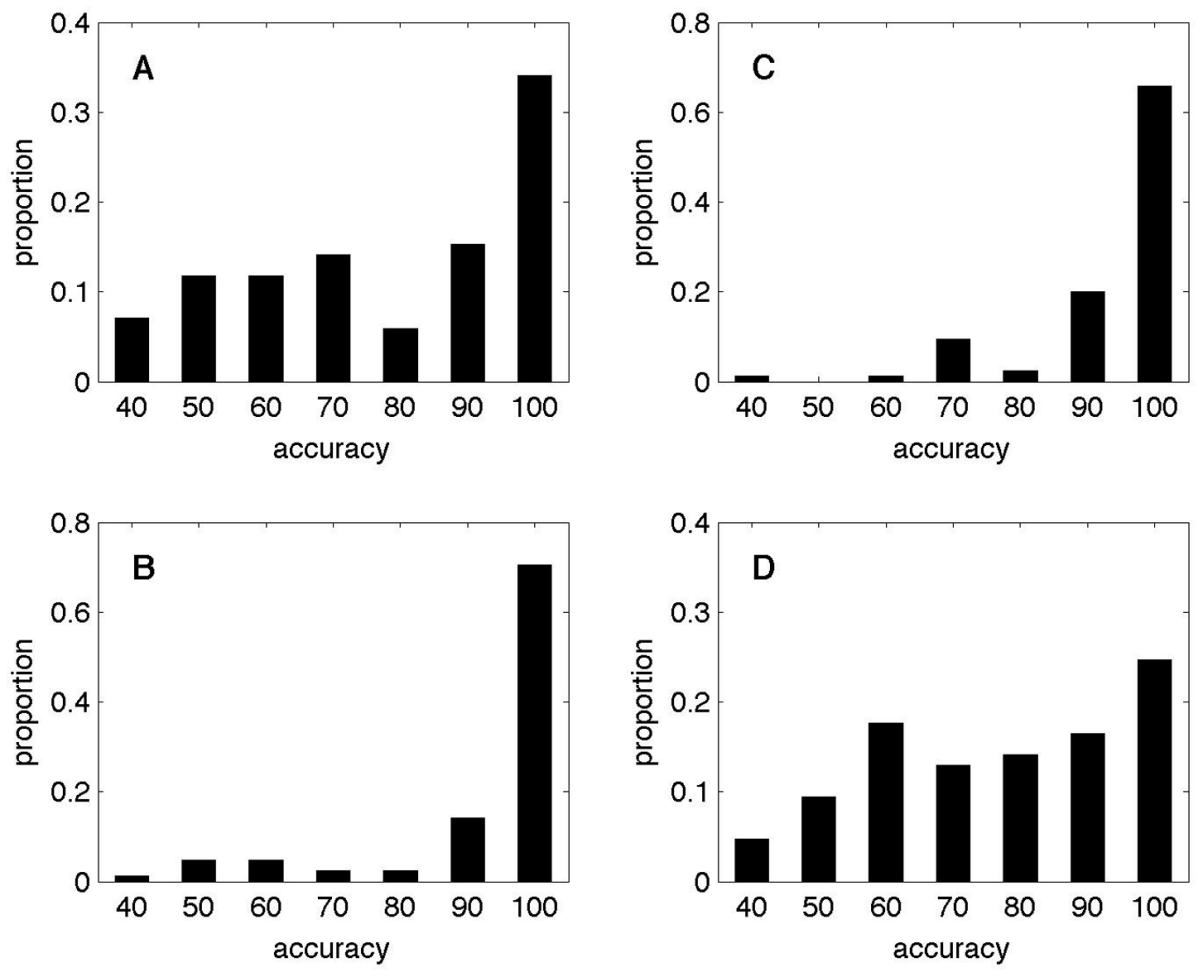
Histogram distribution of user’s (upper panels) and producer’s (lower panels) accuracies for forest change (left) and stable forest (right) categories.

From these samples, I also extracted standard user’s and producer’s accuracies for each category. The user’s accuracy measures the proportion of classified areas as belonging to a category that should be labeled in that category, while the producer’s accuracy measures the proportion of map areas belonging to a category that is classified as belonging to that class, and are related to the to commission and omission errors. Both the user’s and the producer’s accuracies range from about 40 to 100 percent for both classes but the distribution of these errors across classes varies. User’s accuracy for the forest change category is more evenly distributed between 40 and 100 percent although the bulk of the distribution is at the higher end (Figure 3 panel A). The producer’s accuracy, on the other hand, is consistently high for this class as shown in Figure 3 panel B. For the stable forest category, the results are reversed: much higher accuracies are found for the user’s accuracy while a more even distribution of producer’s accuracies exists for this category (Figure 3 panels C and D).

**Figure 4:**
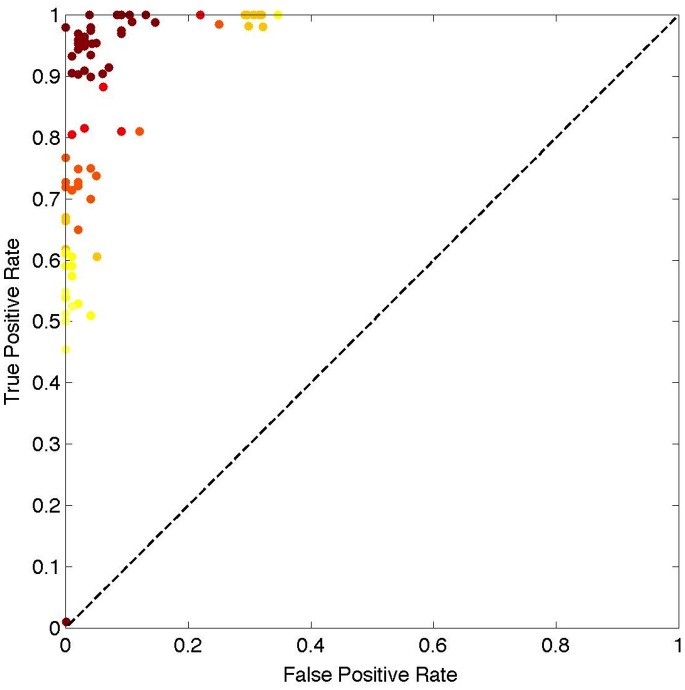
ROC curve showing the distribution of true and false positive rates of all samples used in the analysis. The color range from yellow to dark red is indicative of overall accuracy of the sample, where red reflects high accuracy.

I also defined accuracy based on the rate at which the proposed method could correctly identify forest change (also known as the *True Positive Rate*) and the rate at which it identified forest change when there was none (also known as the *False Positive Rate*) in a Receiver Operator Characteristics (ROC) curve (Figure 4). Several conclusions can be drawn from this analysis. First, the majority of data points lie at the upper left corner of the ROC curve, indicating that the classification results are non-random and the approach produces reliable classification results. Second, the probability of falsely identifying a forest patch as change when there was no change is smaller than the probability of correctly identifying a patch as changed. Third, there is a relationship, albeit an expected one, between overall accuracy – indicated by the color of the dot on Figure 4 – and the amount of true or false positive rate in the classification results.

**Figure 5:**
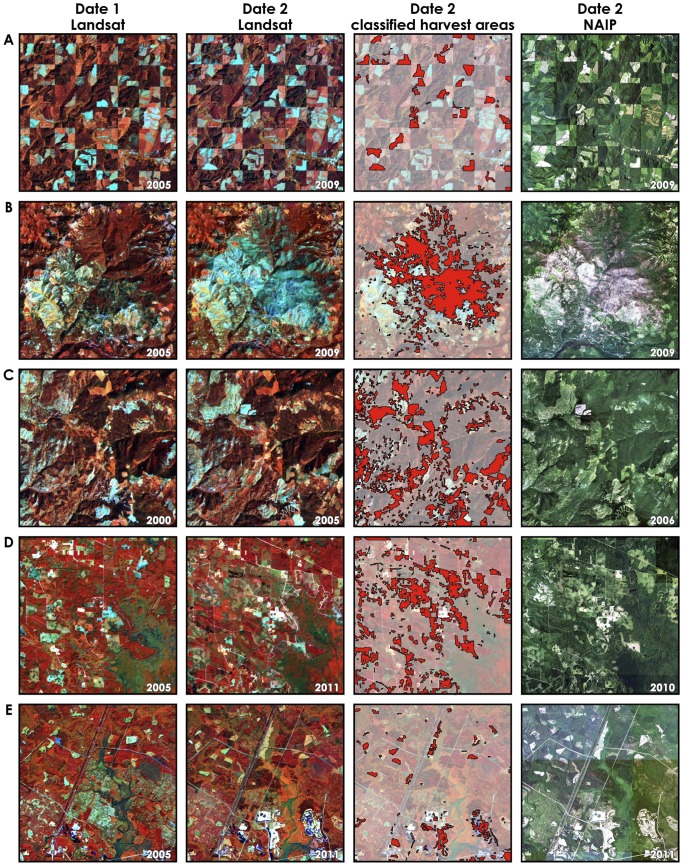
Samples used in the visual assessment of map accuracy. The left two columns are the Landsat images used in the change detection process. The third column is the derived change polygons displayed on the original Landsat images. The last column is NAIP imagery acquired in the year closest to the post-forest disturbance year. The locations are: A. Oregon (path/row 46/30); B. Oregon (path/row 46/30); C. Washington (path/row 44/26); D. Georgia (path/row 17/38); E. South Carolina (path/row 17/38).

**Figure 6:**
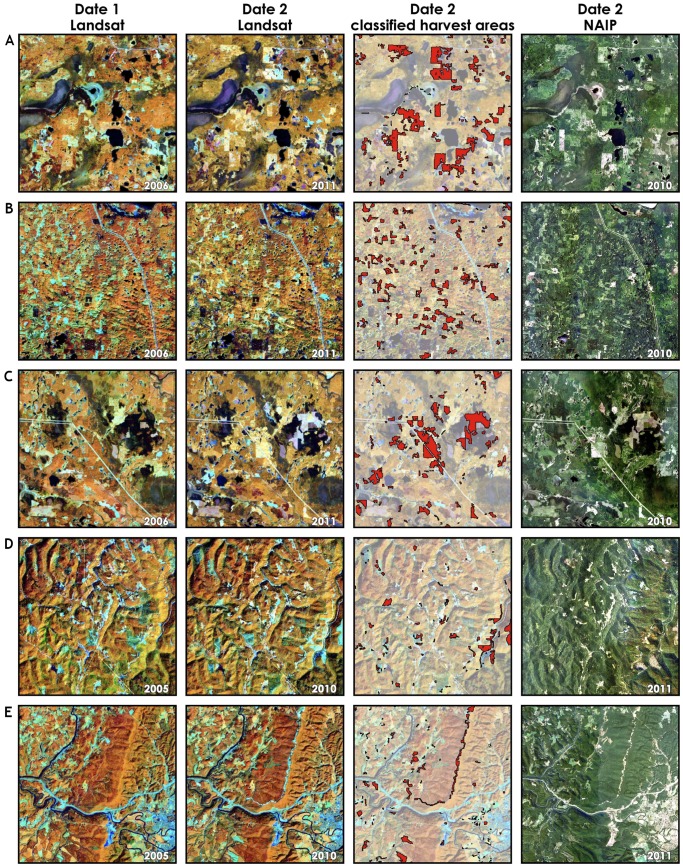
Same as Figure 5 but for different locations: A. Minnesota (path/row 28/27); B. Minnesota (path/row 28/27); C. Minnesota (path/row 28/27); D. West Virginia (path/row 17/33); and E. West Virginia (path/row 17/33).

The final form of accuracy assessment involved visual assessment of the change maps, extracted from a portion of each footprint, against the original Landsat images as well as against the National Agriculture Imagery Program (NAIP) data for the year closest to the change date (Figures 5 and 6). First, in all cases, the derived forest change maps accurately capture the spatial distribution of forest disturbance as evidenced by both Landsat and NAIP imagery interpretation (Figure 5 panels A through E). Second, most errors are confined to commission errors, especially in landscapes with complex terrain (e.g. Figure 5 panels B and C). This finding is also verified quantitatively by the accuracy assessment reported in Figures 2 and 3. Third, non-ideal acquisition dates for image pairs such as an acquisition too early or too late in the season, as well as long periods of time between image dates reduce the reliability of maps derived from the automated procedure. For example, there is a one-month gap (August 3 to September 2) between the acquisition dates of the West Virginia pair (Figure 6 panels D and E). While the automated approach successfully determined harvested conifer stands between the dates, some issues remain due to errors of omission given the leaf-off status of the deciduous stands. With the successful launch of Landsat 8 in early 2013 [62], there will be more opportunities to acquire data during leaf-on seasons as a potential and simple remedy to this issue.

### The Effects of Filtering

**Figure 7:**
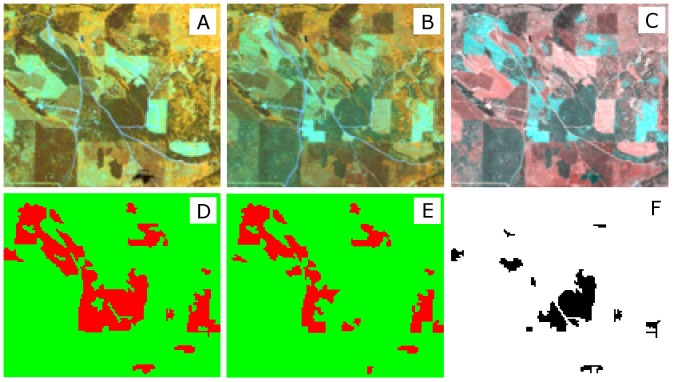
The effects of filtering mislabeled training data on classification accuracy in a forest only landscape in Northwestern Wisconsin. A. pre-harvest image bands 453 as RGB; B. post-harvest image bands 453 as RGB; C. band 5 difference image; D. filtered result; E. unfiltered result; and F. difference between the results.

**Figure 8:**
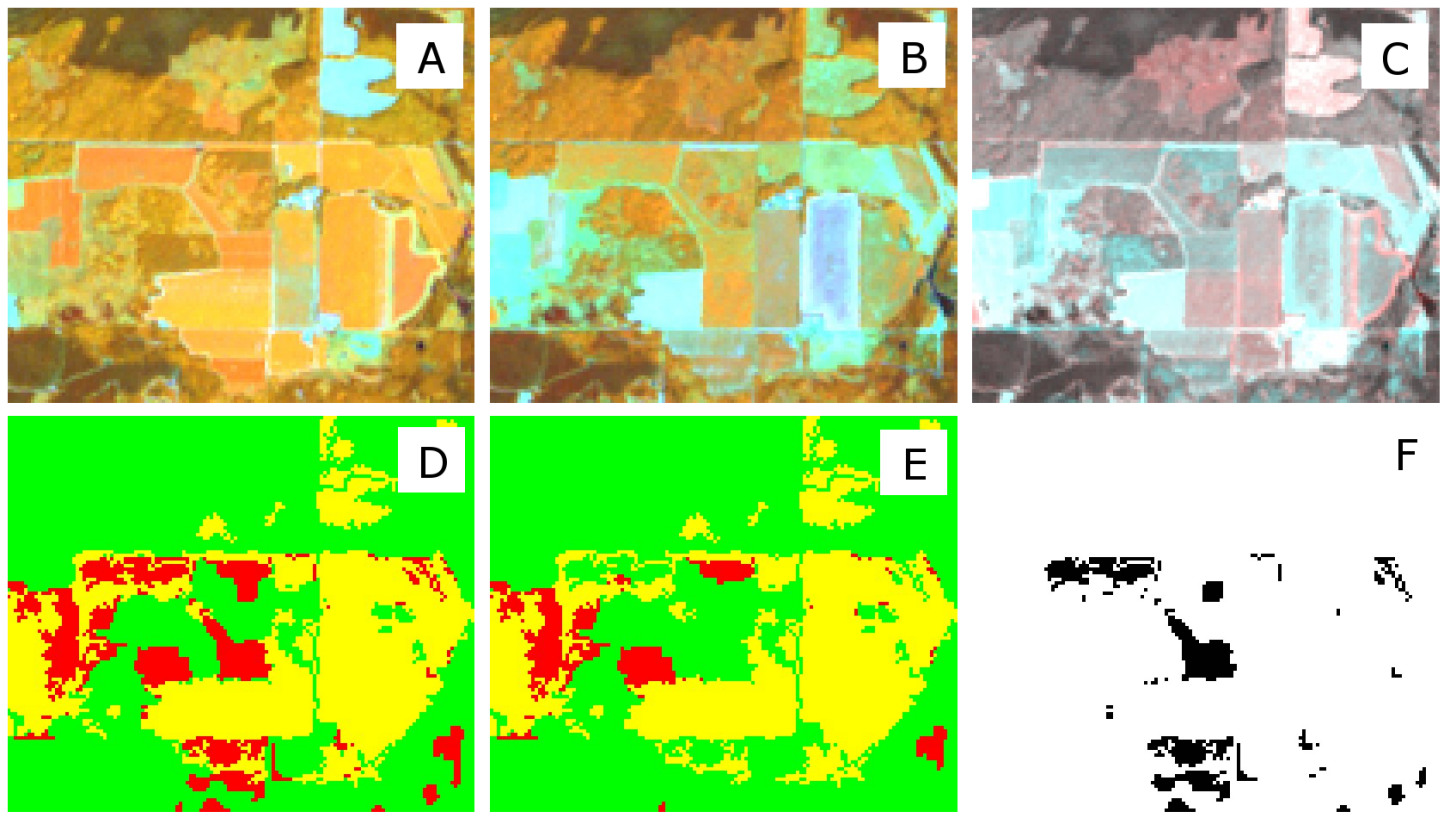
The effects of filtering mislabeled training data on classification accuracy in a forest and agriculture mosaic landscape in Southern Wisconsin. A. pre-harvest image bands 453 as RGB; B. post-harvest image bands 453 as RGB; C. band 5 difference image; D. filtered result; E. unfiltered result; and F. difference between the results.

On average, the filtering process removed 20 percent (20±7%) of the original, automatically extracted training data. The removal of incorrectly-labeled training data significantly improved the classification accuracy (at the 0.05 level of significance using a paired t-test) in many cases. The primary improvements occurred through a reduction of false positives, or areas falsely identified as forest change when there was none as illustrated in Figures 7 and 8. In the first case, a forest-only area composed of both coniferous and deciduous stands targeted for harvest is shown (Figure 7). The changes in the landscape are clearly manifested in both the before- and after-harvest images, as well as in the Band 5 difference image (Figure 7 upper panel). Without filtering, most of the coniferous stands located in the center of the scene are labeled as harvested, although visual inspection of the Landsat images clearly indicates that this is not the case (Figure 7, lower left panel). When this image is classified using the filtered training data, this stand is not longer a part of the forest change category, which is the correct expectation (Figure 7, lower left panel). Finally, the image in the lower right panel highlights the differences between classification results achieved with and without filtering.

In the second case presented in Figure 8, a mixed forested/agriculture landscape is chosen. In this example, the main benefit of removing the incorrectly labeled samples is the reduction of false positives, or areas incorrectly identified as forest disturbance in cropland areas. For example, the tea kettle-shaped agricultural field in the center is classified as forest change with the unfiltered training data, but not with the filtered input. Similarly, the wide, short field in the northwest center was partially classified as forest change, even though it is clearly an agricultural field in the first date (Figure 8, upper and lower panels). Note that filtering does not improve the incorrectly-labeled agricultural area in the center west part of the image. In this case, both the filtered and unfiltered set produce the same incorrect forest change label (Figure 8).

## Discussion

The traditional two-date change detection process in forested areas requires user interaction to define a threshold separating real changes from those caused by a variety of other factors, including seasonality and changes in illumination conditions. When analyzing forest changes over large areas, this user intervention adds significant cost to production. The automated methods proposed here eliminate this user input and are suggested as a simple method to map large areas quickly and efficiently.

However, several requirements must be met for the approach to become an operational methodology. First, the method relies on good geometric correction of images. While all images from the USGS archive are distributed as geometrically corrected and GIS-ready, occasionally there were scenes with shifted coordinates. Of the 85 images analyzed for this work, two were identified as having this problem. Second, the method relies on images acquired during the peak growing period, at least for the deciduous sites. A difference of more than three months causes the false positive rate to increase significantly. While seasonality does not present a significant challenge for detecting disturbance in coniferous forests, leaf-on images are recommended for deciduous areas. Given the improved acquisition strategies and opportunities for more frequent sampling by missions such as Landsat 8 and Sentinel II, this problem may be eliminated in the near future [62].

In areas with persistent cloud cover, the automated change algorithm produces results with large amounts missing data, leading to maps that are not useable. While the recently developed forest change methods that rely on dense time series deal with this issue by replacing data gaps left by clouds and cloud shadows with data from the dense time stack [20], [21], the method described here is not capable of doing this. Dense time stacks are not always available for all regions, which is why the proposed method may be more appropriate in some cases. Imperfect cloud and cloud shadow screening also presents significant challenges to the change detection algorithm described here. The most adverse effect of missed cloud or cloud shadow pixels is that the changes in brightness values are incorrectly labeled as disturbed areas. Third, the current version of the proposed approach does not capture forest regrowth. However, with a few modifications, it would be possible to automatically extract training data for re-establishing forest stands and produce a map of forest recovery. This extension may further allow separation of forest disturbance from permanent land cover change involving forests.

Fourth, as it is currently implemented, the proposed method cannot distinguish forest harvest from natural disturbances or land cover change. The process described here simply looks for and identifies the spectral manifestation of changes on the ground, but does not identify the type of changes. Although changes unrelated to forest disturbance are minimized by using an internal forest mask and filtered training data, the cause of changes on the ground is not reported, in line with many change detection methods. Given modifications to the approach presented in this work to capture forest recovery following a disturbance, it may be possible to further limit areas that have experienced permanent land cover change (e.g. conversion to agriculture or urban, built-up classes). Moreover, because it is relatively fast, the algorithm can produce change maps quickly. This allows users to compare multiple change maps in forested areas, which may in turn reveal the causes of disturbance depending on the pattern, timing, and the nature of the disturbance. Finally, there is a requirement imposed by the number of years between the images that make up the pair. In several cases, forest disturbance occurred immediately after the early image in the pair (pre-disturbance), leaving up to five years for the disturbed area to recover. This led to a reduction in the Band5 reflectance difference that is used as a basis for mapping disturbance. While SVMs are robust towards this form of variation in training data, the limit of a threshold used to separate disturbance from stable forests is quickly reached and may exclude areas with more subtle change signals.

Given these requirements and limitations, what is the utility and benefit of the automated forest change detection method described here? There are several immediate advantages worth mentioning. First, the proposed method does not require any interactive pre-processing involving decision-making by the image analyst. While there are a number of pre-processing steps such as cloud and cloud shadow masking, forest/non-forest delineation, and water/land masking, these are all accomplished automatically using well-tested, well-established algorithms. The only required input is the image pair from the USGS or other archives. These terrain-corrected image sets are provided to the algorithm without any further modification. Second, the algorithm is particularly useful in areas with less-than-ideal temporal coverage. There is no doubt that the growing temporal depth of Landsat and Landsat-like observations provides a unique opportunity for algorithms that operate across a temporal sequence of images [20], [21]. However, due to various acquisition strategies and cloud cover, the temporal record is not growing at the same pace in every region. In fact, as stated in [20], the biggest challenge in applying a dense record of temporal images is constructing an appropriate stack of Landsat imagery, and without it, the accuracy of change detection may diminish precipitously [54], [63], [56].

Another relevant question concerns the processing time required to derive a single change map automatically relative to existing methods. Using a 2010-era computer, each footprint takes about 30 minutes to process. The 85 image pairs tested here required two days of processing time. Given the overall accuracy of the final maps, this quick processing time presents a significant improvement for map-making over large areas. The most time-consuming parts of the process are the SVM classification and image segmentation. Depending on the complexity of the training data, the SVM processing takes somewhere between 5 and 20 minutes with an additional 10 minutes required for the segmentation process. While this may be considered slow, it is necessary to convert the per-pixel classification output to a polygon-based map. The improvement in processing time becomes especially important as the number of images requiring classification increases due to an increase in the size of the area to be mapped or the frequency with which forest change is to be monitored.

Finally, there are several conclusions that can be drawn from examining the patterns of observed errors in the change maps. First, when non-forested areas, especially those involving agricultural land, are mistakenly included in the training data (for example, as a result of imperfect forest/non-forest masking), the mis-classification errors rapidly increase as changes in agricultural cycles are confused with forest change. Inclusion of areas such as agricultural land, shrubland or brush can cause large errors of commission in the forest change class, so a robust method/mask is required to screen out these areas. Nevertheless, the fact that many of these observed errors can be traced to understandable and potentially correctable causes is encouraging.

It is important to note that the filtering procedure may remove samples of a particular class, or of a subset of the classes, to achieve higher accuracy in the remaining classes. While I did not test this hypothesis implicitly, the fact that filtering never decreased accuracy, even in cases with near-perfect (i.e. with very small number of filtered values) training datasets, suggests that filtering does not sacrifice the accuracy of some of the classes to improve overall accuracy.

The reason for improved SVM classification results with the filtered training data was most likely related to the way the SVMs search for an optimal solution to class separation [35]. When the SVM searches for a non-linear decision boundary using kernel functions, the improved training data allow better definition of this boundary by providing more samples to be used in the criteria for class decision. Since easier samples are not generally useful because they are often far from the hyperplane, an improvement in training data is likely to improve the definition of the decision boundary.

## Conclusion

In this paper, I provide a set of procedures that automate the process of image-to-information translation for detection of changes in forested areas. The method relies on the distinct spectral signature associated with forest harvest present in multi-date Landsat imagery, and the ability to automatically capture this signal without user interaction and use it in a robust supervised classification algorithm. The proposed approach can handle incorrectly labeled training data. Although inherently a change detection exercise involving a supervised classification algorithm, the proposed method eliminates the need for manual image interpretation for extracting training data. It is specifically designed to work in situations where dense time stacks of Landsat imagery are not available and the user is forced to use a pair of images over large areas rather than relying on the dense spectral-temporal profile.

The implementation of the automated method is accomplished in several steps: a) pre-processing of pair-wise Landsat images in the form of cloud and cloud shadow masking and excluding all non-forest areas including water and agriculture using masks; b) automated extraction of training data using Landsat shortwave infrared (SWIR) band difference image with local windows; c) automatic removal of incorrectly labeled instances in the training data; d) classification of harvested areas using an implementation of the SVM supervised classification algorithm; and e) per-pixel to per-region translation of classification results using a segmentation analysis.

The requirements for the method to produce appropriate forest change products include peak growing season acquisition of two images that form the pair, accurate masking of forest and non-forested areas, and a robust methodology to screen clouds and cloud shadows. When these conditions are met, the method can be used to produce forest change maps rapidly and with accuracies on par with those delivered by traditional change detection methods. One of the advantages of the proposed approach is the speed: a pair of Landsat images can be processed to create a change map in under an hour. Finally, the automated approach can screen incorrectly labeled instances from the dataset used to train the SVM algorithm, which improves the classification accuracy by reducing commission errors.

When applied to 17 Landsat footprints covering a wide range of forested ecosystems undergoing different rates and amounts of change, the automated method produced forest change maps with overall accuracies in the 80 to 90 percent range based on both quantitative and visual assessment of a large number of samples. The new method is especially useful for forest cover change analysis over very large regions due to the relatively high accuracies, little or no user input required for processing, speed of map production, and the simplicity. Finally, after some modification, the algorithm is also capable of producing maps of forest recovery, presenting a simple tool for complete forest change assessment.
